# Supplementary material for: FORK-seq: replication landscape of the Saccharomyces cerevisiae genome by nanopore sequencing
Source: Genome Biol. 2020 May 26;21:125. doi: 10.1186/s13059-020-02013-3 (PMC7251829; doi:10.1186/s13059-020-02013-3)
Supplement: Supplementary file 1 — Additional file 1 Supplementary tables. This file contains tables describing the samples (T1,T3, T4); mapping statistics related to Fig. 1 (T2) and a summary of initiation event clustering results (T5). [file 13059_2020_2013_MOESM1_ESM.pdf]

Sample ID	dTTP	BrdUTP
Run ID	160809_MN18874_FAD19767	160809_MN17490_FAD19641
Library protocol	2D-MAP006 low input	2D-MAP006 low input
DNA used for library (ng)	120	100
Flowcell	R9	R9
MinKNOW version	1.0.2.0	1.0.2.0
Basecaller version	2.40.17	2.40.17
Workflow	2D RNN for NSK007	2D RNN for NSK007
Metrichor run ID	113694	113693
Number of reads	114750	77100
Number of "pass" reads	20583	10328
Number of "fail" reads	94167	66772
% mapped	60.0	49.2

Table T1: Detailed information about *in vitro* template sequencing runs. The "% mapped" is the percentage of reads mapped by BWA MEM after Metrichor basecalling.

	dTTP			BrdUTP		
	pass	fail	all	pass	fail	all
Number of reads	20583	94167	114750	10328	66772	77100
% of pass reads			17.9			13.4
TP/CP pairs (2D reads)	20583	47030	67613	10328	33471	43799
% of 2D reads	100.0	49.9	58.9	100.0	50.1	56.8
TP and/or CP unmapped	293	32338	32631	113	25414	25527
TP/CP pairs, at least one mapped	20317	23814	44131	10254	13634	23888
TP/CP pairs, both mapped	20290	14692	34982	10215	8057	18272
TP mapped on F	10305	23357	33662	4305	11932	16237
TP mapped on R	10009	29544	39553	5946	17850	23796
CP mapped on F	9987	7879	17866	5914	4636	10550
CP mapped on R	10306	8013	18319	4304	4604	8908
TP unmapped	269	41266	41535	77	36990	37067
CP unmapped	290	31138	31428	110	24231	24341
TP + CP	41166	141197	182363	20656	100243	120899
TP + CP mapped	40607	68793	109400	20469	39022	59491
TP + CP unmapped	559	72404	72963	187	61221	61408
% mapped	98.6	48.7	60.0	99.1	38.9	49.2

Table T2: Mapping summary for the *in vitro* templates. TP, template strand (first strand to enter the pore), CP, complement strand (second strand). The mapping was performed independently for the two strands, using BWA MEM. F, forward plasmid strand (primer extended), R, reverse strand (parental).

Sample ID	Thy_R1	BrdU_R1	Thy_R2	BrdU_R2	BrdU_9	BrdU_38	BrdU_69
Run ID	MN19358_FAH14273	MN17490_FAH14319	FAH58492_MN1747	FAH54284_MN19358	MN17479_FAK06634	MN17273_FAK06596	FAH58543_MN19358
Sequencing date	20170808	20170821	20180214	20180213	20181010	20181010	20180220
DNA used for library (ng)	1507	1502	2022	2200	1499	1505	1941
Flowcell	R9.5	R9.5	R9.4.1	R9.4.1	R9.4.1	R9.4.1	R9.4.1
MinKNOW version	1.7.10	1.7.14	1.10.23	1.10.23	'1.14.1	'1.14.1	1.10.23
Albacore version	1.2.3	1.2.3	2.1.10	2.1.10	2.3.3	2.3.3	2.1.10
Number of reads	92928	502122	93697	606184	161954	145595	367216
% Tombo + RepNano	62.1	54.3	87.9	59.7	78.0	70.4	83.4
BrdU % (mass spec)	-	-	0	91	9	38	69

Table T3: Detailed information about yeast training sample sequencing runs. Sequencing date is in YYYYMMDD format. The "% Tombo + RepNano" is the percentage of reads successfully processed by Tombo and RepNano.

Sample ID	pulse_R1_1	pulse_R1_2	pulse_R1_3	pulse_R2_1	pulse_R2_2	pulse_R2_3
Run ID	FAH59201_MN17490	MN17490_FAH68914	MN18874_FAH68992	FAH58519_MN18874	MN19040_FAH69011	MN19358_FAH66366
Sequencing date	20180226	20180416	20180416	20180226	20180416	20180416
DNA used for library (ng)	2000	1518	1518	1999	1736	1736
Flowcell	R9.4.1	R9.4.1	R9.4.1	R9.4.1	R9.4.1	R9.4.1
MinKNOW version	1.10.23	1.11.5	1.11.5	1.10.23	1.11.5	1.11.5
Albacore version	2.1.10	2.1.10	2.1.10	2.1.10	2.1.10	2.1.10
Number of reads	275030	255596	388174	354300	224498	191502
% Tombo + RepNano	78.7	84.2	86.3	83.8	86.1	84.9

Table T4: Detailed information about pulse-chase sample sequencing runs.

	number of initiation events					number of clusters		
	all	events ORI $\leq$ 2kb	events ORI >2kb	cluster median ORI $\leq$ 2kb	cluster median ORI >2kb	all	cluster median ORI $\leq$ 2kb	cluster median ORI >2kb
d=1	362	106	256	106	256	362	106	256
d=2-4	373	259	114	263	110	140	96	44
d=5-12	1060	1012	48	1036	24	134	130	4
d>13	3169	3118	51	3169	0	143	143	0
d>1	4602	4389	213	4468	134	417	369	48
total	4964	4495	469	4574	390	779	475	304

Table T5: Summary of the clustering of initiation events. Left, initiation events; right, clusters. For each indicated cluster dimension d, the given numbers from left to right are those of: all events; events close to ($\leq$ 2 kb) or far from (> 2 kb) a known origin (ORI); events whose cluster median point is close to ($\leq$ 2 kb) or far from (> 2 kb) a known origin (ORI); all clusters of indicated dimension; clusters of indicated dimension whose median point is close to ($\leq$ 2 kb) or far from (> 2 kb) a known origin (ORI).
