# Supplementary material for: FORK-seq: replication landscape of the Saccharomyces cerevisiae genome by nanopore sequencing
Source: Genome Biol. 2020 May 26;21:125. doi: 10.1186/s13059-020-02013-3 (PMC7251829; doi:10.1186/s13059-020-02013-3)
Supplement: Supplementary file 3 — Additional file 3 Review history. [file 13059_2020_2013_MOESM3_ESM.docx]

Review History

**First round of review**

**Reviewer 1**

**Were you able to assess all statistics in the manuscript, including the appropriateness of statistical tests used?**

Yes: I checked the statistics

**Were you able to directly test the methods?**

No

**Comments to author:**

The authors presented a study that used nanopore sequencing to study DNA replication (which they refer to as FORK-seq) in S. cerevisiae. Since nanopore sequencing generates ionic current signals when a DNA molecule translocates through the pore, it is in principle possible to differentiate natural nucleotides (A, C, G, T) from unnatural nucleotides (in this case, BrdU). They found that although most initiation events clustered at known origins, 7 % occurred at dispersed locations undetectable by population methods. They propose a model in which eukaryotic chromosome replication combines clustered initiation at efficient sites and dispersed initiation at inefficient sites.

1. I am surprised that so many reads are failed, and the mapped rates are too low, although they are not 1D data. Some explanations are necessary. Note that they used 2D sequencing, which is completely obsolete nowadays (due to patent infringement issues) so the data is of less practical value to the community; nevertheless, if the data is truly of high quality, that can still be useful for algorithm development of DNA replication software tools. There are quite a few red flags on how the data are processed and I wonder whether these explain the high failure rate and low mapping ratio: for example, they used Metrichor (ONT) for base calling (with acknowledgement that "the presence of BrdU may affect basecalling"), used BWA MEM for alignment (yet minimap2 is now commonly used), etc. But in any case, 2D is supposed to be much better than 1D, yet the quality of the data appears highly questionable. Indeed, "pass" reads was low for BrdUTP (13%) than for dTTP (18%), and these ratios are so low that typically I would have declared an experimental failure if this is the result from a nanopore run.

2. The introduction section did not actually describe background in a comprehensive manner. In particular, it completely ignored multiple biorxiv papers and published papers essentially reporting the same/similar approaches to study DNA replication or study DNA modification. They did cite a recent Nature Methods application with the exact words "Consistent with a recent study [7]," or "Together with the recent study of Muller et al [7]," without even saying anything about what was actually done in the reference 7. Frankly I do not see any realistic differences or improvements that this paper has over reference 7, and in fact the "200 bp resolution" in the current study bothered me because I cannot seem to see where it really comes from.

3. Page 8: it would be better to provide the number of compared initiation sites, and the exact number of detected initiation sites which coincided with. Using "most initiation sites" does not provide accurate information.

3. To demonstrate the usage of RepNano, it would be better to use it to detect initiation sites on both thymidine-only control and BrdU samples to see the false positive rates. Since several software tools are already developed for this purpose of examining DNA replication, it would be also important to compare to existing tools (for example, the same reference 7 that they have mentioned in Introduction), to show advantages of their own software tools or to show the differences in conclusion drawn from different software tools.

4. No clear how they generate prediction target during training process of CNN. They basically incorporated "methods" in the Results section, and therefore omitted essential details on the computational tool. I feel that it is important to both describe the experimental data and the computational tool developed for the experimental data, especially if the authors believe that their computational tool has advantages over existing tools that are already published.

5. Why 96bp window size? Any optimization or evidence for this size threshold. I recognize that they use this approach to avoid to call individual bases but estimates BrdU content for sliding windows; I think this makes sense and can be an alternative approach than TM that they also used, but where does this magic number come from?

6. I feel that the description of initiation and termination events, and the discussion on distribution of initiation events are the highlights of the paper, and I applaud the authors in doing these analysis. I am however concerned about over-interpretation of the data, not only due to the low quality of data per se, but also the stochastic nature of computational predictions (which can be caused by biases in the data). I think some part of the Results should be really Discussions instead; for example, events that are far from known OriDB origin could simply be incorrect computational predictions, or be due to experimental biases (DNA break for example). I should say that the statement such as "a model in which 93% of initiation events occur at specific positions that share a consensus sequence motif and coincide with previously known origins" are very strong statement, that needs to be backed by lots of high-quality data and multiple replicate experiments, possibly even in different strains. Indeed the reference 7 says 20% of all initiation events may occur dispersed through the yeast genome which is very different from 7% reported here.

7. Figures are of low quality. It is hard to see the detail, for example in Figure 2e.

8. It would be helpful to use line types rather than sole line colors in Figures. This is just a suggestion to improve readability of the presentation.

9. As I already mentioned previously, things like "We have developed a novel method, termed FORK-seq" is an over-statement. They need to actually discuss existing literature and show what are differences in their experimental and computational approaches.

10. Finally in the Discussion they explained the statement "replicative track start and initiation event detection by FORK-seq is 200 nucleotides." I cannot seem to understand this statement and where the 200bp comes from. They mentioned that the precision of estimation of BrdU amount along DNA is in the range of 100 bp, based on transition from low to high BrdU content. Then mention the slight discordance between CNN and TM, with median distance between true and detected starting points of 120 and 136 bp using CNN and TM. Then mention clusters common to two replicates. Then this magic number 200bp suddenly pops up from nowhere.

11. I like the discussion of FORK-seq and D-NAscent differences. This is a good piece of information, but as I said earlier it would be important to show technical details of their approach. The "Neural network architecture" in Methods is very short, and even shorter than what's in the Results section.

**Reviewer 2**

**Were you able to assess all statistics in the manuscript, including the appropriateness of statistical tests used?**

Yes: No additional statistical review is required

**Were you able to directly test the methods?**

No

**Comments to author:**

This method article from Hyrien and colleagues reports the development of a novel assay called FORK-seq that uses nanopore sequencing to map replication origins at 200 nucleotide resolution. To illustrate the potential of this assay, individual initiation events were mapped in the genome of budding yeast and positioned these sites relative to known replication origins. To this end, live yeast cells were pulse-labeled with BrdU and the position of nascent DNA was determined by sequencing individual DNA molecules with a MinION system and assigning the position of BrdU-labeled tracks with two independent computational methods (CNN and TM). Using this approach, the authors identified and oriented nearly 60 thousand individual BrdU tracks, from which they derived the position of ~5000 individual initiation events. Remarkably, 93% of these sites clustered within 2 kb of each other at loci containing a confirmed replication origin. They also identified sporadic initiation sites that were not previously identified by other assays and that do not contain typical ARS consensus sequences. Interestingly, they also mapped a large number of replication termination sites, which correspond to broader regions than initiation sites.

The data presented in this study are consistent with the conclusions of a recent study from the group of Conrad Nieduszynski (Müller et al, 2019) using a similar approach, but at a much lower resolution. Together, these studies demonstrate the potential of nanopore sequencing to monitor DNA replication in eukaryotic cells. Indeed, unlike population-based assays such as BrdU-IP-seq, these single-molecule approaches give access to cell-to-cell variations that are not accessible to population-based assays. Moreover, FORK-seq and other nanopore sequencing assays are applicable to virtually any organism incorporating BrdU. It is therefore likely that nanopore sequencing will soon become the new standard for the analysis of DNA replication at the level of individual molecules.

Overall, the data presented in this manuscript are of very high quality and the methodology is clearly described. This method article should be of a wide general interests to the readers of Genome Biology. However, important issues need to be addressed prior to publication.

Specific issues:

1. The methodology used to detect individual forks is robust and convincing, but the strategy used to derive the position of individual initiation events is less clear. Initiation sites were defined in this study as the midpoints between diverging forks. Since 58000 forks were detected and oriented, one would have expected the assignment of up to 29000 origin positions. However, only 20% these sites were reported and the reason(s) for this discrepancy should be discussed. In particular, the length distribution of sequenced molecules (10-140 kb, p.8) should be displayed. Are BrdU-containing fragments shorter than unlabeled molecules?

2. The authors used OriDB as to position annotated origins, but also refer to a list of 235 high-confidence origins (ref #20), which is not included in OriDB (p. 7). The authors should provide a complete list of reference origins used in this study and indicate what fraction of this list overlaps with FORK-seq origins.

3. Current literature on origin usage often refer to clusters of initiation sites to define multiple initiation events occurring next to each other on the same DNA molecule. Here, the authors use the term 'cluster' in a different way. They should therefore stress the fact that their clusters do not refer to initiation zones, for clarity.

4. The fact that FORK-seq origins cluster within 2 kb is impressive, considering that these sites are defined as the midpoint between diverging forks. This high degree of clustering implies that sister forks progress at the same speed and indicates that the method used to position them is extremely reproducible. Are FORK-seq origins always equally distributed around ARS consensus sequences, as illustrated in Figure 3 or do some origins show a bias, as predicted by the fact that MCMs can be pushed away from the ACS by transcription (Gros, 2015, Mol Cell 60, 797)?.

5. Figure 2f and 3a: The position of early and late/dubious origins should be indicated with different colors.

6. The authors refer to Eaton et al. (ref #20) for the impact of nucleosome occupancy on replication initiation but they should also cite Berbenetz et al. (2010) PLoS Genet 6, e1001092

7. Similarities and differences between FORK-seq and D-Nascent, the assay recently published by the Nieduszynski lab, are discussed in a balanced manner. However, several limitations should be addressed. The comparison of FORK-seq and D-Nascent output indicates that the level of BrdU substitution is a key parameter of the assay. However, the use of a thymidine-auxotroph strain to increase substitution could be a problem as these cells could have perturbed intracellular dNTP pools, which may affect both fork speed and origin usage. When applied to mammalian cells, the technique may be limited by the length of the sequenced DNA molecules. It is likely that these limitations will be quickly circumvented, but these issues need to be discussed.

8. Acknowledgements: … sequencing platform of I2BC.

**Authors Response**

**Point-by-point responses to the reviewers’ comments:**

*Authors Answers to the Editors' requests and the Reviewer Reports:*

*We thank the two Referees for their careful reading of this manuscript. We have provided below a point-by-point response to each of their comments.*

*We also have included additional analyses at the request of the Editors who asked for "more demonstration of how FORK-seq is able to perform better than the D-NAscent methodology of Müller et al", and "how its higher resolution gives researchers and users some practical benefits and leads to biological insights".*

*1. We compared the BrdU detection tool of D-NAscent with our RepNano methods (Fig. S5; Results page 6 of 23; Discussion page 12 of 23). We demonstrate that RepNano strongly outperforms D-NAscent for precise measurement of BrdU incorporation and establishment of replication fork direction (RFD). See also answers to points 2 and 3b of referee 1.*

*2. We demonstrate that we better detect late origin firing than Müller et al (Fig. S11). Our BrdU labeling method gives unbiased representation of replication tracks regardless of replication time and is free of cell synchronization artifacts, which Müller et al cannot guarantee due to the use of synchronized cells.*

*3. In addition to the presence of an ACS motif, we looked at ORC and MCM binding using published ChIP-seq data (Fig. 5d-f). Clustered but not sporadic origins are associated with strong ORC and MCM peaks. Sporadic origins inefficiently recruit ORC and MCM, which we associate to their lack of strong enrichment of ACS motifs. ACS enrichment and ORC and MCM binding were not analyzed in Müller et al.*

*4. We have analyzed replication termination in more detail (Fig. 6, Fig. S7d, Fig. S8, Fig S9). Termination occurs at locations and relative efficiencies previously reported (Fachinetti et al 2010, McGuffee et al 2013), but we demonstrate that it is even more dispersive than previously thought, with 855 out of 4,485 termination events mapped at previously undetected positions. There was no analysis of termination in Müller et al.*

*5. We further demonstrate that FORK-seq detects events inaccessible by cell-population methods. We show that 11% and 18% of individual initiation and termination events, respectively, are inevitably missed in cell-population approaches because they occur in regions that on average sustain a majority of termination and initiation events, respectively. A higher proportion of sporadic than clustered events fall in this category (Fig. S9).*

*6. We demonstrate that the difference between the proportion of individual initiation and termination events within a segment is equal to the slope of the RFD profile established from the population of all oriented BrdU tracks (Fig. 7). Therefore, there is a high consistency between the full extent of cell-to-cell heterogeneity revealed by single-molecule analysis and the statistical average of fork direction observed in the cell population. Points 5 & 6 were not addressed in Müller et al as this requires analysis of termination as well as initiation events.*

Reviewer #1:

*We thank this reviewer for his thorough criticism. Although some criticisms appeared to us less justified than others, we did our best to answer all of them. We believe this has overall resulted in a much improved and stronger manuscript.*

The authors presented a study that used nanopore sequencing to study DNA replication (which they refer to as FORK-seq) in S. cerevisiae. Since nanopore sequencing generates ionic current signals when a DNA molecule translocates through the pore, it is in principle possible to differentiate natural nucleotides (A, C, G, T) from unnatural nucleotides (in this case, BrdU). They found that although most initiation events clustered at known origins, 7 % occurred at dispersed locations undetectable by population methods. They propose a model in which eukaryotic chromosome replication combines clustered initiation at efficient sites and dispersed initiation at inefficient sites.

1. I am surprised that so many reads are failed, and the mapped rates are too low, although they are not 1D data. Some explanations are necessary. Note that they used 2D sequencing, which is completely obsolete nowadays (due to patent infringement issues) so the data is of less practical value to the community; nevertheless, if the data is truly of high quality, that can still be useful for algorithm development of DNA replication software tools. There are quite a few red flags on how the data are processed and I wonder whether these explain the high failure rate and low mapping ratio: for example, they used Metrichor (ONT) for base calling (with acknowledgement that "the presence of BrdU may affect basecalling"), used BWA MEM for alignment (yet minimap2 is now commonly used), etc. But in any case, 2D is supposed to be much better than 1D, yet the quality of the data appears highly questionable. Indeed, "pass" reads was low for BrdUTP (13%) than for dTTP (18%), and these ratios are so low that typically I would have declared an experimental failure if this is the result from a nanopore run.

*The referee refers here to the plasmid experiments shown on Figure 1 and notes that some of the software used for these test plasmid experiments are now outdated. However, this did not prevent us to detect a specific signature of BrdU incorporation. Furthermore, the referee failed to note the 99% mapping rate of "passed" reads in Figure 1 and the 54 - 88% mapping rate of yeast genomic DNA independently called with the most recent software. The rate of mapping in Figure 1 does not impact any of our conclusions regarding yeast DNA replication. Actually, Figure 1 could be deleted without affecting the rest of the manuscript. We chose to maintain it following the Editor's comments on a former submission: "We think that the ability of Nanopore to call modified bases has been underexplored so far, so this is an interesting aspect of this work."*

2. The introduction section did not actually describe background in a comprehensive manner. In particular, it completely ignored multiple biorxiv papers and published papers essentially reporting the same/similar approaches to study DNA replication or study DNA modification.

*Apart from Müller et al 2019 (ref 7; now ref 11) and our own bioRxiv preprint (Hennion et al 2018; ref 10), we found only one bioRxiv preprint, Georgieva et al 2019, addressing the use of nanopore sequencing to study DNA replication. This preprint reported the detection of multiple thymidine analogues in synthetic templates and of IdU incorporated through a complete cell cycle in mouse cells but, in contrast to ref 10 & 11,, it did not use this to map DNA replication. We have now quoted these three references in the introduction.*

*As to other DNA modifications, we did quote reports of cytosine methylation detection (ref 12, 13 in former submission) in the discussion. They are now quoted in the introduction (ref 7,8 in this revision). Studies of other RNA or DNA modifications that did not address DNA replication are too numerous to quote, but in this revised version we have included in the introduction the review by Xu and Seki, "Recent advances in the detection of base modifications using the Nanopore sequencer ", published in Journal of Human Genetics (Oct 11) after our original submission (ref 9).*

They did cite a recent Nature Methods application with the exact words "Consistent with a recent study [7]," or "Together with the recent study of Muller et al [7]," without even saying anything about what was actually done in the reference 7. Frankly I do not see any realistic differences or improvements that this paper has over reference 7, and in fact the "200 bp resolution" in the current study bothered me because I cannot seem to see where it really comes from.

*The differences between our method and that described in ref 7 (now ref 11) were described in detail in the Discussion paragraphs starting at "FORK-seq and D-NAscent are similar in principle but differ in details..." and "FORK-seq precision can be assessed at different levels of analysis..." and in Figure S7 (now S6). The referee him/herself declared (point 11) " I like the discussion of FORK-seq and D-NAscent differences. This is a good piece of information,....". We therefore understand that his/her dissatisfaction, that we "did not say anything about what was done" in reference 7, only concerns the introduction. We have rewritten an introductory paragraph where we now quote and describe in more detail ref 7 (now ref 11), the bioRxiv preprints by Georgieva et al (ref 12) and by Hennion et al (ref 10), and the review by Xu and Seki (ref 9).*

*Please note that in response to point 3b below we have now tried to use the D-NAscent detection tools on our reads and found that they performed much less well than our CNN and TM tools, as detailed in the Results. We modified the Discussion paragraph accordingly. "(...) The BrdU signal obtained by D-NAscent software detection was located at similar positions to RepNano but was weaker, precluding automatic BrdU track detection and orientation and it did not lead to a valid RFD profile. The number of B calls was about 4 times lower in D-NAscent than in TM: in the highly substituted sample, TM and D-Nascent called 10.3% and 2.6% of all T sites, respectively. This represents a significant difference between the D-NAscent and FORK-seq computational methodologies. The other significant difference between the D-NAscent and FORK-seq experiments is the replication labeling strategy (...)".*

*Finally, we do not understand how this referee cannot see where the "200 bp resolution ...really comes from". S/he has not provided any objective refutation of the data shown in Figure S7 (now S6). How can s/he state that this 200 bp number "suddenly pops up from nowhere" (point 10) ? This round number is an easy-to-read, conservative estimate based on the data shown in Figure S7 (now S6) and their extensive comments in the text. Nevertheless, we have tried to improve the presentation of this point. We have included a new section entitled "FORK-seq precision" in the Results, which clearly demonstrate that the technical precision of track start detection is better than 200 nucleotides. We have also added a paragraph at the end of the Results section "Distribution of initiation events in S. cerevisiae" (page 8 of 23) where we show that the variability of origin detection (as defined by a cluster's median point) between biological replicates, which reflects both the technical imprecision of FORK-seq and the true biological dispersion of initiation events, is 219 bp, consistent with the technical precision. Finally, we have more concisely rewritten the relevant Discussion paragraph (page 11 of 23). Please also see our reply to point 10.*

3a. Page 8: it would be better to provide the number of compared initiation sites, and the exact number of detected initiation sites which coincided with. Using "most initiation sites" does not provide accurate information.

*Accurate numbers are provided in the description of Figure 3 and recapitulated in the section "A two-process model for S. cerevisiae genome replication initiation".*

3b. To demonstrate the usage of RepNano, it would be better to use it to detect initiation sites on both thymidine-only control and BrdU samples to see the false positive rates.

*Following this suggestion we found ZERO false positives in the thymidine-only control. This is now mentioned in the text (Page 5 of 23, five lines from bottom). Looking at the shape of replication initiation signals (e.g. Figures 2c, 3b and S6ab), it was in fact very hard to imagine how noise could create such coherent signal patterns that match the very specific BrdU incorporation pattern observed at origins, extending over a length (several kb) that is much larger than the window size used for BrdU incorporation estimates. The referee's suggestion (point 6) that "events far from known oriDB origins could simply be incorrect computational predictions" is therefore refuted.*

Since several software tools are already developed for this purpose of examining DNA replication, it would be also important to compare to existing tools (for example, the same reference 7 that they have mentioned in Introduction), to show advantages of their own software tools or to show the differences in conclusion drawn from different software tools.

*We have now used the D-NAscent software to analyse our reads and compare with our CNN and TM software (Fig S5, text Page 6 of 21). Although BrdU was detected at the same positions, the signal was weaker. Using the same algorithm as for CNN or TM signals, most D-NAscent tracks could be neither detected nor oriented even after trying to optimize the detection parameters (Fig. S5). Consequently, the RFD profile assembled from the oriented D-NAscent tracks was of very poor quality, showing very low correlation coefficients to the four RFD profiles shown in Figure 2f: FORK-seq TM, 0.15; FORK-seq CNN, 0.13; OK-seq MCM869, 0.12; OK-seq ligase mutant, 0.11. Therefore, our CNN and TM tools clearly outperform the D-NAscent tools. This is now explained in the Results (page 6) and commented in the Discussion (page 12).*

4. No clear how they generate prediction target during training process of CNN. They basically incorporated "methods" in the Results section, and therefore omitted essential details on the computational tool. I feel that it is important to both describe the experimental data and the computational tool developed for the experimental data, especially if the authors believe that their computational tool has advantages over existing tools that are already published.

*We agree with this referee that it is fundamental to describe both experimental data and computational developments. We had omitted to reference in the Results the Materials and Methods paragraph describing the CNN construction and training, giving the impression that important information about CNN architecture and learning procedure was missing. We have now corrected this omission. Furthermore, we now provide more information about the CNN learning procedure in this paragraph and a new Figure S4 illustrating in more detail the adopted architecture of RepNano’s CNN.*

5. Why 96bp window size? Any optimization or evidence for this size threshold. I recognize that they use this approach to avoid to call individual bases but estimates BrdU content for sliding windows; I think this makes sense and can be an alternative approach than TM that they also used, but where does this magic number come from?

*As correctly mentioned by this referee, our CNN architecture was designed so as to directly estimate BrdU content in a given window thus avoiding to call individual BrdU bases. This approach entails dimensionality reduction from the window size to 1. This is achieved by the action of several layers each performing a fraction of the required dimensionality reduction. As is common practice, we first use two layers of maxpooling over 4 consecutive values that each reduces the size of the vectors by a factor of 4, so that their combined action is a 16-fold dimensionality reduction. This implies the input window size to be a multiple of 16. Moreover it must contain enough informative transitions to reliably train the neural network. We evaluated using the TM method that 514 (12.5%) of the 4,096 pentamer-to-pentamer transitions were informative for BrdU incorporation. The CNN window size was therefore chosen to be 96 bp as this is a multiple of 16, containing 14 informative transitions on average, which we reasoned was enough. This is now mentioned in the caption of Figure S4 and in the Material and methods section "Neural network architecture" .*

6. I feel that the description of initiation and termination events, and the discussion on distribution of initiation events are the highlights of the paper, and I applaud the authors in doing these analysis. I am however concerned about over-interpretation of the data, not only due to the low quality of data per se, but also the stochastic nature of computational predictions (which can be caused by biases in the data). I think some part of the Results should be really Discussions instead; for example, events that are far from known OriDB origin could simply be incorrect computational predictions, or be due to experimental biases (DNA break for example).

*As detailed in our response to point 3b, the concern with incorrect computational predictions is refuted by the fact that we found zero false-positive signals in the thymidine-only controls.*

*As to the suggestion that such events could be due to DNA breaks, we have to say that we do not understand how DNA breaks - or other undefined "experimental biases" - could artificially create the type of signals shown on Figures 2, 3 and S7. Did the referee mean that BrdU incorporation during break repair could produce exactly similar signals to those observed at known origins? Nucleotide or base excision or single strand break repair would each result in much shorter tracks (tens of nucleotides at best). Double strand break repair may produce longer BrdU tracks but synthesis would be unidirectional. In contrast, events far from known oriDB origins show bidirectional synthesis and are exactly similar in mapping quality and other criteria to those observed at previously reported origins. Finally, the amount of non-canonical initiation we detect is equivalent to 20-30 initiation events per cell cycle, orders of magnitude larger than the estimated number of double strand breaks per cycle. Apart from DNA replication and DNA repair, we are not aware of any "experimental biases" that could result in such significant incorporation of BrdU in DNA. This is now mentioned page 7 of 23, at the end of the section "Detection of individual initiation and termination events".*

I should say that the statement such as "a model in which 93% of initiation events occur at specific positions that share a consensus sequence motif and coincide with previously known origins" are very strong statement, that needs to be backed by lots of high-quality data and multiple replicate experiments, possibly even in different strains. Indeed the reference 7 says 20% of all initiation events may occur dispersed through the yeast genome which is very different from 7% reported here.

*The reasons for the different estimates of the prevalence of dispersive initiation by D-NAscent vs. FORK-seq have been extensively discussed, as explained in our answer to point 2. As Müller et al computed a z-score of BrdU incorporation by 2kb windows, their resolution cannot be better than 2 kb. We demonstrated on Figure S7 (now S6) that our resolution is in the order of 200 bp, which is obviously much better. Please have a look at Figure S11b for a direct comparison of D-NAscent and FORK-seq resolution. The simplest and most likely explanation for the 20% vs. 7% discrepancy is just the difference in the precision of the two methods, although we acknowledge in the Discussion that we cannot rule out some effect of the different genetic backgrounds and synchronization and labelling procedures.*

*Although this referee raised doubts about "the low quality of the data", referee 2 declared that "the data presented in this manuscript are of very high quality and the methodology is clearly described". We mentioned that we sequenced two biological replicates of the pulse-chase experiment with a good correlation between the corresponding RFD profiles. This and the above arguments indeed confirm a far better quality of our data than those of Müller et al, who only reported one origin mapping experiment. We therefore do not think that multiplying replicate experiments in different strains is justified.*

7. Figures are of low quality. It is hard to see the detail, for example in Figure 2e.

*The online submission process automatically generate a pdf where the figures are indeed of low quality. The high quality figures can be downloaded directly from the website (a link is included in the pdf next to each figure). We anyway increased the size of the numbers in Figure 2e that were indeed too small.*

8. It would be helpful to use line types rather than sole line colors in Figures. This is just a suggestion to improve readability of the presentation.

*Done.*

9. As I already mentioned previously, things like "We have developed a novel method, termed FORK-seq" is an over-statement. They need to actually discuss existing literature and show what are differences in their experimental and computational approaches.

*The differences in experimental and computational approaches between FORK-seq and D-NAscent are extensively discussed in the Discussion, as already explained in our answers to point 2. Please note that referee 2 found that " Similarities and differences between FORK-seq and D-Nascent, the assay recently published by the Nieduszynski lab, are discussed in a balanced manner." In addition to our previous text, we now clearly show that our CNN and TM BrdU detection tools outperform the D-NAscent detection tool (Fig. S5; Results page 6 of 23; Discussion page 12 of 23). Differences in both computational methodologies and replication labeling strategies are discussed in the revised version.*

10. Finally in the Discussion they explained the statement "replicative track start and initiation event detection by FORK-seq is 200 nucleotides." I cannot seem to understand this statement and where the 200bp comes from. They mentioned that the precision of estimation of BrdU amount along DNA is in the range of 100 bp, based on transition from low to high BrdU content. Then mention the slight discordance between CNN and TM, with median distance between true and detected starting points of 120 and 136 bp using CNN and TM. Then mention clusters common to two replicates. Then this magic number 200bp suddenly pops up from nowhere.

*See our answers to point 2. We tried to analyze 2 sources of imprecision in our data: the BrdU track start detection (technical imprecision, Fig S6a-c, ~100-140bp) and the variability of origin position (as defined by the median point of a cluster of initiation events) between biological replicate experiments (Fig S6d, ~220bp). As the latter depends on both the technical imprecision of the technique and the true biological dispersion of initiation events and/or inequality of fork progression around origins, the replicate variability is consistent with the measured technical precision. We therefore propose an easy-to-read, round number, conservative estimate of FORK-seq precision at 200 bp.*

11. I like the discussion of FORK-seq and D-NAscent differences. This is a good piece of information, but as I said earlier it would be important to show technical details of their approach. The "Neural network architecture" in Methods is very short, and even shorter than what's in the Results section.

*We have rewritten the paragraph ‘CNN learning’ in the Materials and Methods section to ensure that sufficient information was provided to allow reproducibility. Furthermore, we now provide a new Figure S4 to illustrate the paragraph ‘Neural network architecture’ where focus is now on the information flow from sequence-aligned nanopore current shifts to BrdU incorporation estimate.*

Reviewer #2:

*We thank this referee for his/her positive comments and constructive criticism. We have provided an answer to each of his/her points.*

This method article from Hyrien and colleagues reports the development of a novel assay called FORK-seq that uses nanopore sequencing to map replication origins at 200 nucleotide resolution. To illustrate the potential of this assay, individual initiation events were mapped in the genome of budding yeast and positioned these sites relative to known replication origins. To this end, live yeast cells were pulse-labeled with BrdU and the position of nascent DNA was determined by sequencing individual DNA molecules with a MinION system and assigning the position of BrdU-labeled tracks with two independent computational methods (CNN and TM). Using this approach, the authors identified and oriented nearly 60 thousand individual BrdU tracks, from which they derived the position of ~5000 individual initiation events. Remarkably, 93% of these sites clustered within 2 kb of each other at loci containing a confirmed replication origin. They also identified sporadic initiation sites that were not previously identified by other assays and that do not contain typical ARS consensus sequences. Interestingly, they also mapped a large number of replication termination sites, which correspond to broader regions than initiation sites.

The data presented in this study are consistent with the conclusions of a recent study from the group of Conrad Nieduszynski (Muller et al, 2019) using a similar approach, but at a much lower resolution. Together, these studies demonstrate the potential of nanopore sequencing to monitor DNA replication in eukaryotic cells. Indeed, unlike population-based assays such as BrdU-IP-seq, these single-molecule approaches give access to cell-to-cell variations that are not accessible to population-based assays. Moreover, FORK-seq and other nanopore sequencing assays are applicable to virtually any organism incorporating BrdU. It is therefore likely that nanopore sequencing will soon become the new standard for the analysis of DNA replication at the level of individual molecules.

Overall, the data presented in this manuscript are of very high quality and the methodology is clearly described. This method article should be of a wide general interests to the readers of Genome Biology. However, important issues need to be addressed prior to publication.

Specific issues:

1. The methodology used to detect individual forks is robust and convincing, but the strategy used to derive the position of individual initiation events is less clear. Initiation sites were defined in this study as the midpoints between diverging forks. Since 58000 forks were detected and oriented, one would have expected the assignment of up to 29000 origin positions. However, only 20% these sites were reported and the reason(s) for this discrepancy should be discussed. In particular, the length distribution of sequenced molecules (10-140 kb, p.8) should be displayed. Are BrdU-containing fragments shorter than unlabeled molecules?

*Many reads contain only one fork, therefore one expects less than 29000 initiation or termination events. The length distribution of all molecules, molecules with ≥1 fork, and molecules with ≥2 forks is now shown on Figure S10 (referenced in the text page 11 of 23). As explained in the Figure legend, BrdU tract-containing fragments are in fact longer than unlabeled molecules, because of the size-threshold required for BrdU-tract detection which eliminates the shortest reads. Furthermore, molecules with at least one initiation or termination event are even longer, because the probability that a read contains >1 BrdU-tract increases with read size, since converging or diverging forks are necessarily spaced apart on replicating DNA by some non-null distance.*

2. The authors used OriDB as to position annotated origins, but also refer to a list of 235 high-confidence origins (ref #20), which is not included in OriDB (p. 7). The authors should provide a complete list of reference origins used in this study and indicate what fraction of this list overlaps with FORK-seq origins.

*We only used the 253 high-confidence ORC positions from ref 20 to derive the ARS consensus sequence matrix, not as a list of origin positions. The list of OriDB origins is provided with the analysis scripts. Among the OriDB origins, 57% (473/829) overlap with FORK-seq origins; this fraction is 79% (326/410) for the confirmed OriDB origins, 44% (95/216) for the likely origins and 26% (52/203) for the dubious ones. The overlap was defined after resizing all objects to 2kb. This information is now stated in the section “Distribution of initiation events in S. cerevisiae” (page 8 of 23).*

3. Current literature on origin usage often refer to clusters of initiation sites to define multiple initiation events occurring next to each other on the same DNA molecule. Here, the authors use the term 'cluster' in a different way. They should therefore stress the fact that their clusters do not refer to initiation zones, for clarity.

*This distinction is now stated at the beginning of the section “Distribution of initiation events in S. cerevisiae” (page 7 of 23).*

4. The fact that FORK-seq origins cluster within 2 kb is impressive, considering that these sites are defined as the midpoint between diverging forks. This high degree of clustering implies that sister forks progress at the same speed and indicates that the method used to position them is extremely reproducible. Are FORK-seq origins always equally distributed around ARS consensus sequences, as illustrated in Figure 3 or do some origins show a bias, as predicted by the fact that MCMs can be pushed away from the ACS by transcription (Gros, 2015, Mol Cell 60, 797)?.

*This is a very interesting question but we cannot fully address it at present. To start investigating this, we carefully examined the five ARSs (ARS 305, 447, 1303, 1414, 1415) shown in detail in Gros et al (Figure 4 A and 6) to undergo a shift in MCM or Okazaki fragment distribution in the rat1 mutant at restrictive temperature, but please note that no such shift was observed at permissive temperature. Consistently, we did not observe any asymmetric distribution of the left and right forks with respect to the ACS (see Figure A for referee 2 at the end of this document). The median distance between the midpoints of diverging forks and the ACS was < 140 bp in all cases, except for ARS447 where the vicinity of another likely ARS and the low number of mapped initiation events precluded any definitive conclusion. A similar situation is observed for the FORK-seq origins of chromosome 3 and 8 (see Figure B for referee 2). A more complete study may be performed but this would require additional data such as the transcription status of genes around origins in our strain and a high-resolution mapping of MCMs (Bedalov, BioRxiv) that is not yet publicly available.*

5. Figure 2f and 3a: The position of early and late/dubious origins should be indicated with different colors.

*Done.*

6. The authors refer to Eaton et al. (ref #20) for the impact of nucleosome occupancy on replication initiation but they should also cite Berbenetz et al. (2010) PLoS Genet 6, e1001092

*Ref 24,25 are now cited together in the Results section "Characterisation of the sporadic initiation events", page 9 of 23.*

7. Similarities and differences between FORK-seq and D-Nascent, the assay recently published by the Nieduszynski lab, are discussed in a balanced manner. However, several limitations should be addressed. The comparison of FORK-seq and D-Nascent output indicates that the level of BrdU substitution is a key parameter of the assay. However, the use of a thymidine-auxotroph strain to increase substitution could be a problem as these cells could have perturbed intracellular dNTP pools, which may affect both fork speed and origin usage.

*The relevant discussion paragraph (page 13 of 23, middle) has been modified to include this point.*

When applied to mammalian cells, the technique may be limited by the length of the sequenced DNA molecules. It is likely that these limitations will be quickly circumvented, but these issues need to be discussed.

*The last discussion paragraph (page 13 of 23) has been modified to include this point.*

8. Acknowledgements: sequencing platform of I2BC.

*Done.*

**Second round of review**

**Reviewer 2**

The authors have properly addressed all the issues raised by both reviewers and have significantly improved the manuscript. In my opinion, it is now suitable for publication in Genome Biology. I have no doubt that this method represents a major advance relative to the D-NAscent assay and that it will be very useful to the DNA replication community.
